# Supplementary material for: Adhesion strength, cell packing density and cell surface buckling in pericellular matrix-mediated tissue cohesion
Source: Development. 2025 Aug 26;152(16):dev204663. doi: 10.1242/dev.204663 (PMC12448319; doi:10.1242/dev.204663)
Supplement: Supplementary information [file develop-152-204663-s1.pdf]

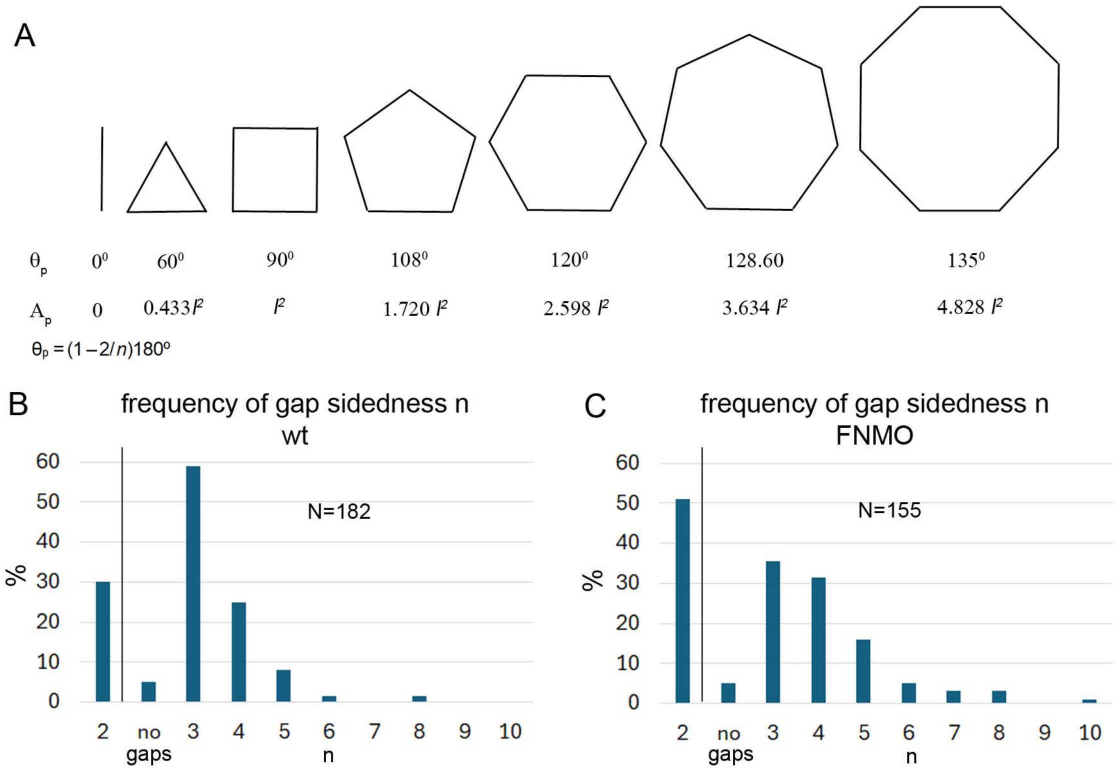

Fig. S1. Gap side numbers. (A) Regular polygons, unit side length *l*, polygon angle θ_p, area A_p in multiples of l^2. (B,C) Frequencies of gaps with n sides, normal (B) and FN-depleted PM (C). Normally, 3-sided gaps are most frequent, but gaps with up to 8 sides occur. In FN morphants, gaps are larger and thus more likely to fuse, increasing the fraction of gaps with $n > 3$. Fraction of 2-sided gaps per contact, as isolated "bubbles" between two cells, become abundant upon FN depletion. n, number of gaps; from 24 and 30 TEM sections, respectively.

Development • Supplementary information

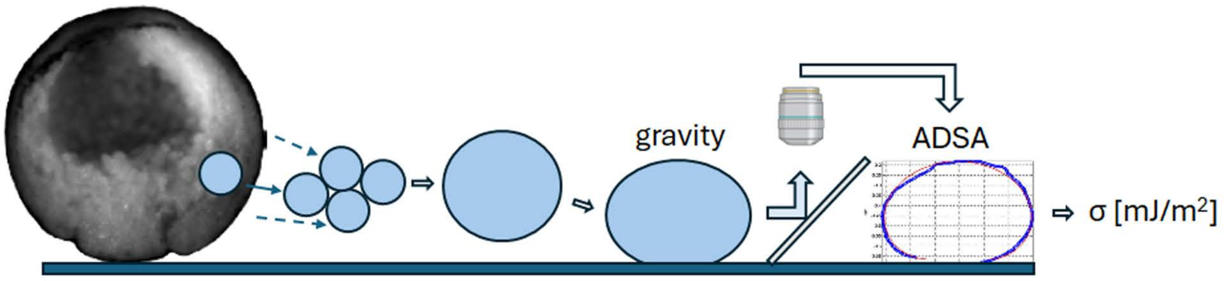

Fig. S2. Measurement of tissue surface tension. PM tissue (blue circle) from gastrula (left) is explanted and combined with explants from several other embryos. The explants coalesce into a single spherical aggregate. On a non-adhesive surface, the aggregate assumes a drop shape under gravity. It is viewed in a 45° mirror for its contour to be segmented by the ADSA program (blue outline in right diagram). ADSA uses the Laplace equation to fit a theoretical outline (red) to the observed contour (blue) and calculates surface tension σ. See Materials and Methods for more details and references.

Development • Supplementary information

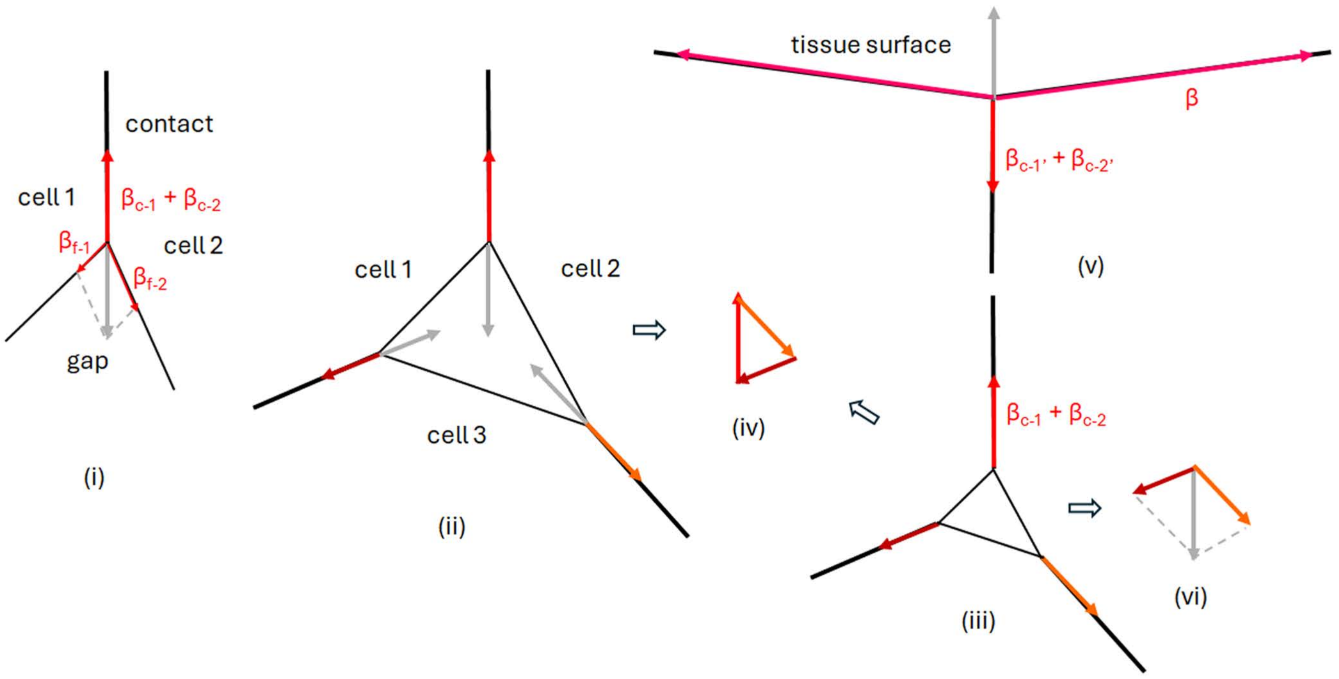

Fig. S3. Tension balances at gaps when tensions vary between different contacts or different free surfaces. (i) Tension equilibrium at gap corners. Added tensions $\beta_{c\text{-}1}$, $\beta_{c\text{-}2}$ of the two cells at a contact (bold red) (in main text, β_c per cell is used) are balanced by the resultant (grey) of gap surface tension $\beta_{f\text{-}1}$, $\beta_{f\text{-}2}$ (light red). At equilibrium, the balanced tensions at gap corners must also balance each other for gap stability (ii, iii). Tensions at contacts represented as vectors (shades of red) must add up to zero (iv), which for geometrically similar gaps does not depend on gap size (compare (ii) and (iii)). Gaps are also in tension equilibrium with the tissue surface (v), and e.g. the resultant (grey) of cortical tensions β at the surface (red) is balanced by a respective resultant at the gap (vi).

Development • Supplementary information

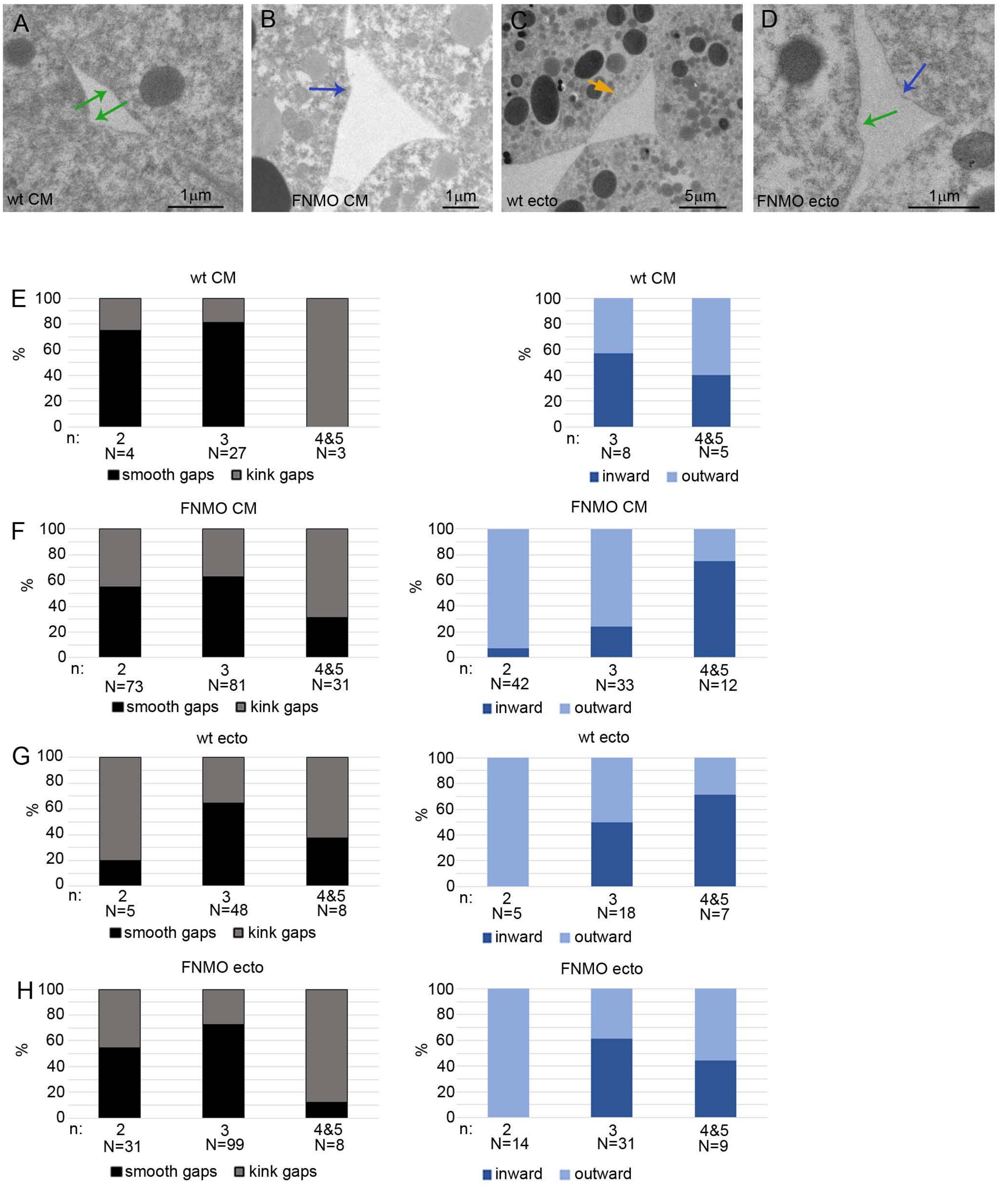

Fig. S4. Kinked gap sides in chordamesoderm and neural ectoderm. (A-D) Examples of gap surface shapes. Normal (A) and FN-depleted chordamesoder (B), normal (C) and FN-depleted neural ectoderm (D) with outward pointing (green arrows) and inward pointing (blue arrows) kinks and S-shaped surface (yellow arrow). (E-H) Statistics for (A-D), from 11 normal and 13 FN-depleted TEM sections each. Left, percentage of gaps with no kinks (black), at least one kinked side (grey) in 2-sided, 3-sided and 4-5-sided gaps. Right, percentage of kinks pointing outward (light blue) or inward (dark blue) in 2-sided, 3-sided and 4-5-sided gaps. N, number of gaps or gap sides examined.

Development • Supplementary information

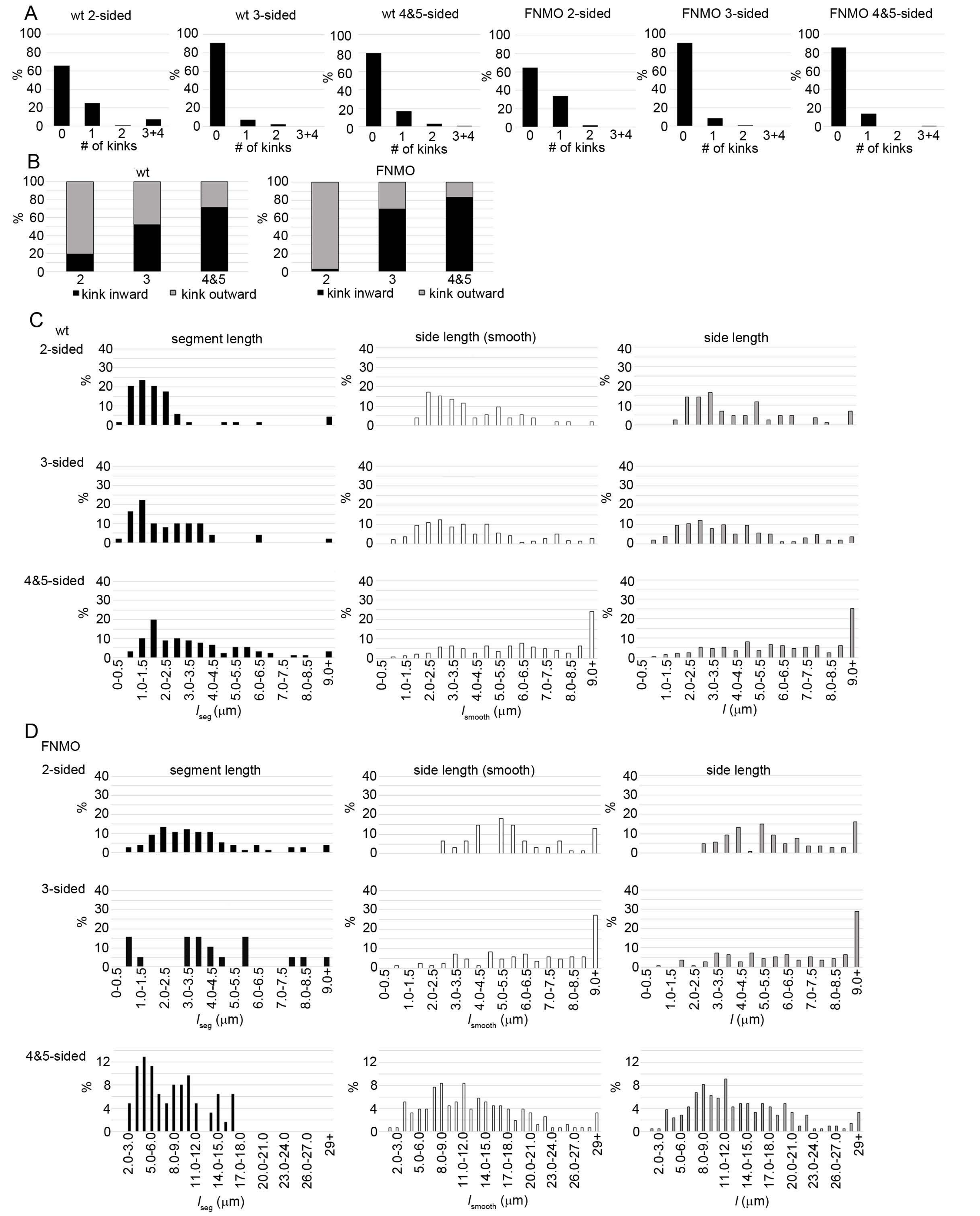

Fig. S5. Characteristics of gaps in PM. (A) Frequency of gap sides with 0 to 4 kinks in normal or FN-depleted PM, in 2- to 5-sided gaps. (B) Fraction of kinks pointing into gap (inward) or into cell (outward) in normal or FN-depleted PM, in 2- to 5-sided gaps. 19 TEM sections from normal and 30 sections from FN-depleted PM. (C,D) Frequencies of segment lengths l_{seg} (left) compared to lengths of non-kinked, smooth gap sides l_{smooth} (middle) and total side lengths l (right), in normal (C) and FN-depleted PM (D), from 2-sided, 3-sided and 4- to 5-sided gaps in each case. 21 TEM sections for normal, 28 for FN-depleted PM.

Development • Supplementary information

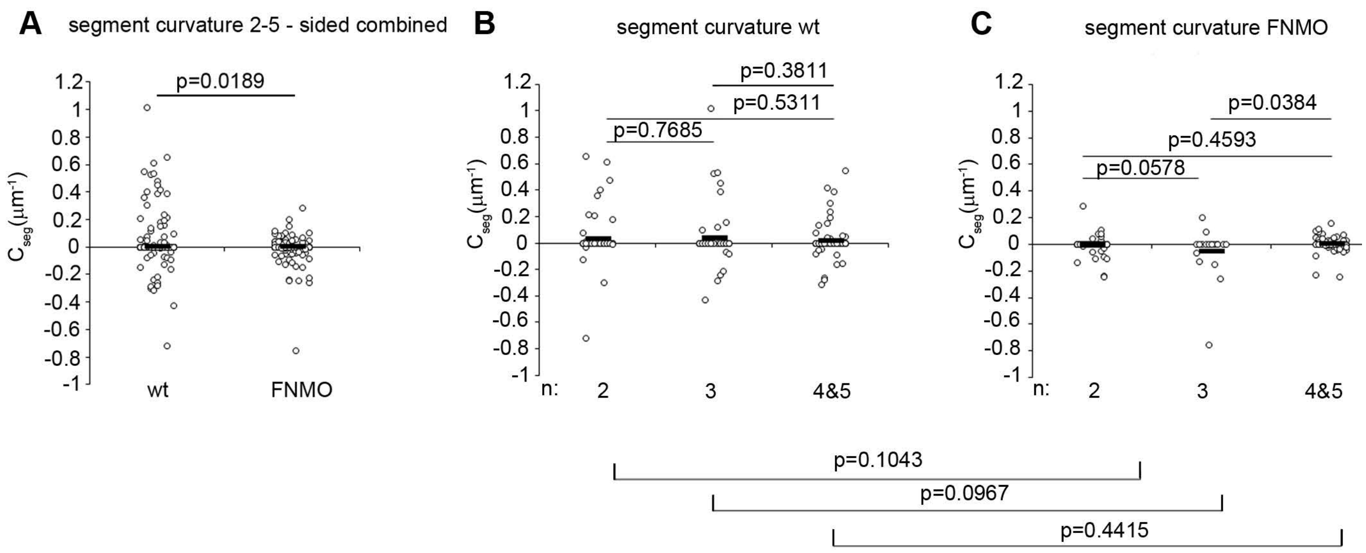

Fig. S6. Curvatures of gap side segments. Values for normal and FN-depleted PM (21 and 28 sections) combined (A) and sorted for *n* (B). Segment curvature C_{seg} does depend on FN or *n*. Averages, black bars; p, significance.

Development • Supplementary information

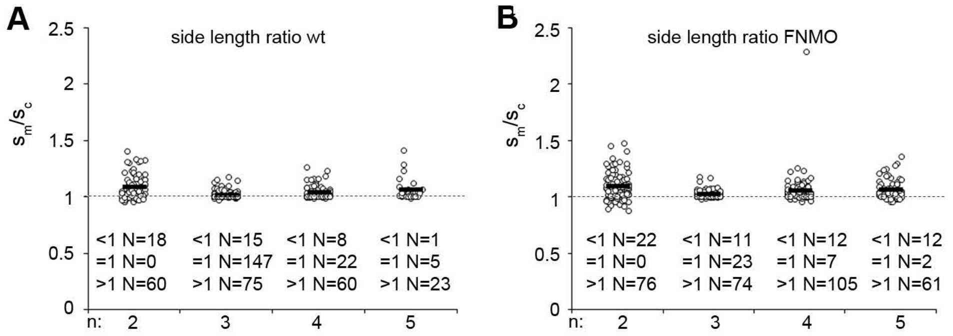

Fig. S7. Ratio of measured/calculated contour lengths, S_m/S_c, for different *n* of normal (A) and FN-depleted PM (B), from 14, 12, 6, 6, 13, 17, 11, 11 sections (left to right). N, number of ratios < 1, 1, and > 1 (see Figure 3K,L). Circle segments calculated as $S_c = \pi(l/\sin\theta_c)(\theta_c/180°)$ with θ_c the angle between l and the cell surface (see Fig. 3J) and $S_c = S_m$ for $\theta_c = 0°$.

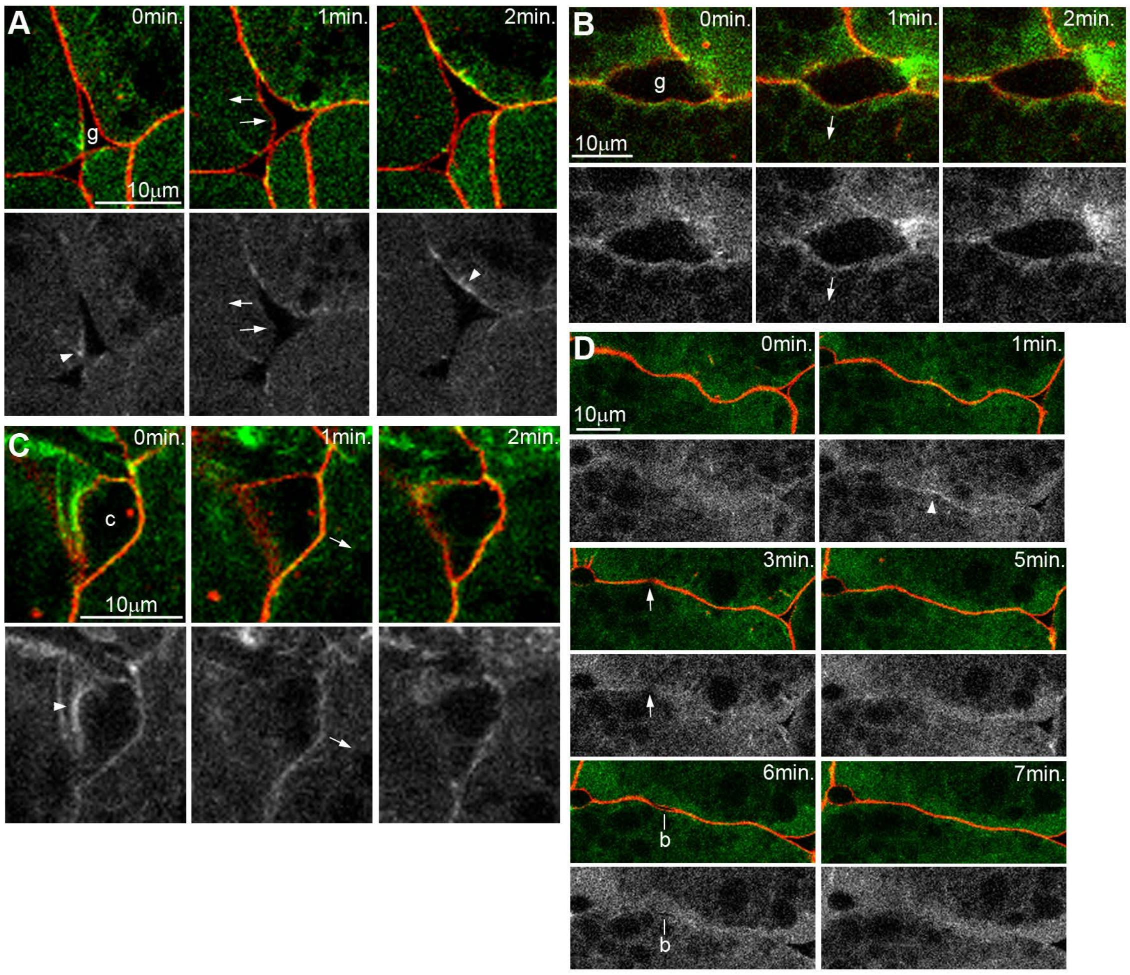

Fig. S8. F-actin and cell surface shapes in PCM explants, with cell membranes labeled with mb-RFP (red) and F-actin with LifeAct-GFP (green). (A,B) Transient, 1-minute kinks (arrows) at gap (g) surfaces. Short cortical F-actin pulses (arrowheads) away from kinked site. (C,D) Kinked (arrows, C,D) or wavy (D) cell-cell contacts, not consistently related to local F-actin condensations (arrowheads). c, part of cell with endocytosed membrane (red dots). Wavy contact in (D) appears stretched out as right-hand 3-sided gap shifts to the right. 2-sided gap (b) forms transiently. 2 movies from 2 independent experiments.

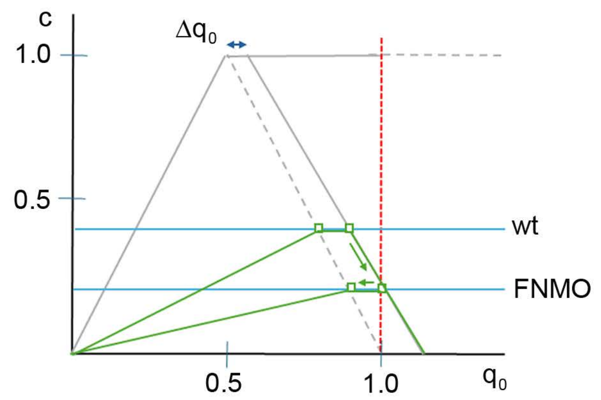

Fig. S9. PCM-mediated adhesion. Contact abundance c as function of q_0 as in Fig.7B (grey lines), but with fraction of PCM being non-adhesive. Initial increase of c is slower, the plateau of compression-dependent interdigitation and the descending branch are reached at higher q_0, but q_0 would be the same for normal or FN-depleted PM as without non-adhesive PCM patches (squares on solid descending line). In the extreme case, the slopes of the curves for normal and morphant PM (green) would be proportional to the reduction of c, i.e. FN knockdown would proportionally increase the fraction of non-adhesive PCM, and the curves would intersect the normal or FN morphant lines (blue) when they reach the plateau (squares on dashed line): contact and hence gap size is fully determined by the fraction of non-adhesive PCM. Various degrees of compression up to the level seen in PM without non-adhesive patches can be achieved by moving along the horizontal plateau lines (green). As in non-patched PM, FN depletion could also lead to an increase in q_0 to reduce c to the observed value (oblique green arrow). Lastly, Δq_0 reflects both the stiffness and area expansion of the PCM, and increasing PCM stiffness in FN morphants, i.e. lowering Δq_0 and thus shifting the descending line in the plot could move the intersection point to lower q_0 (horizontal green arrow). This would help to avoid $q_0 > 1$, a condition where stubs would have to be compressed before interdigitation. Blue lines, c of normal (wt) and FN-depleted (FNMO) PM intersect with c-q_0 curves. Intersection beyond $q_0 = 1$ (red dashed line) implies stubs are compressed before interdigitation.

Table S1. Morpholinos used in experiments

**Morpholino**	**Sequence (5'-3')**	**Nanograms injected per blastomere**	**Efficiency (% reduction of protein)**
C-cadherin[1,2]	CCACCGTCCCGAACAGAAGCCTCAT	20	65
Fibronectin (xFN1)[3,4]	CGCTCTGGAGACTATAAAAGCCAAT	20	63
Fibronectin (xFN2)[3,4]	CGCATTTTTCAAACGCTCTGAAGAC	20	63
xSyn-4.1[5,6]	GCACAAACAGCAGGGTCGGACTCAT	20	75
xSyn-4.2[5,6]	CTAAAAGCAGCAGGAGGCGATTCAT	20	75

Targets (first column) of previously characterized morpholinos (numbers indicate references), sequences (second column), amount injected (third column) and efficiencies as a percentage reduction of protein levels (see references) (fourth column).

References: [1]Ninomiya et al. (2012); [2]David et al. (2014); [3]Davidson et al. (2006); [4]Nagel and Winklbauer (2018); [5]Muñoz et al. (2006); [6]Zhang et al. (2016).

Development • Supplementary information

Supplementary Materials and Methods

In our capillarity-based model the binding energy released upon the interaction of adhesion factors is completely turned into adhesion tension

$B/2 = \Gamma/2$ (Eqn3 in main text).

With this, and by using $\beta_f - \Gamma/2 = \beta_f - B/2$ instead of β_c, Eqn1 and 2 that describe tension differences and ratios at the surface and at interstitial gaps of biphasic tissues can be written

$\sigma = (\beta - \beta_f) + B/2$ (S1a), $\cos\theta = (\beta_f - B/2)/\beta$ (S1b),

$\sigma_i = B/2$ (S2a), $\cos\theta_i = (\beta_f - B/2)/\beta_f$ (S2b).

In the elasto-capillary model, we mark parameters that occur in both models with a prime, '. Binding energy B changes into B' in complex ways. Thus, more stub-stub interfaces per contact area after compression (see Fig. 7A) allows in principle for more binding interactions, but on the other hand, stretching stubs proximo-distally with respect to the membrane could alter the number and strength of accessible binding sites per stub in unforeseeable ways. Also, lateral adhesion between compressed yet non-interdigitating stubs may become significant. Our model implies a membrane-proximal to distal asymmetry of stubs, which in its simplest form would be one of shape, consistent with a proximo-distal elasticity gradient as for example in the endothelial glycocalyx (Kabedev and Lobaskin, 2018). Cone-shaped stubs could fit to each other well when interdigitating but would require strong deformation to attach laterally. Any compression-induced lateral binding energy of stubs would have to be subtracted from that released upon interdigitation to obtain B'. Adhesion tension Γ', compressive elastic energy W_{com} and bending elastic energy W_b are all derived from B' released at interdigitational PCM-PCM interaction, in ratios that minimize the total free energy but are not considered here further. B' is released in contacts only, generating there an adhesion tension Γ' and elastic energies $W_{com\text{-}c}$ and $W_{b\text{-}c}$. $W_{b\text{-}c}$ varies with local curvature, and for unevenly curved segments we take the average $W_{b\text{-}c\text{-}av}$. The overall elastic energy per area of PCM layer in a contact is then $W_c = W_{com\text{-}c} + W_{b\text{-}c\text{-}av}$. While Γ' remains restricted to contacts, surface pressure Π_{crit} is in equilibrium within the continuous PCM layer and compressive energy $W_{com\text{-}g}$ but also bending energy $W_{b\text{-}g\text{-}av}$ on gap surfaces are produced by PCM interpenetration in contacts, i.e., the release of binding energy in contacts is partially used to build elastic energy $W_g = W_{com\text{-}g} + W_{b\text{-}g\text{-}av}$ in gaps. In contacts, B', Γ' and W_c are shared between two cells, and per cell

$B'/2 = \Gamma'/2 + W_c/2 + W_g$ (Eqn 4 in main text).

The addition of elastic energy terms $W_c/2$ and W_g changes Eqn S1 and S2, and we determine how the parameters in these equations are transformed. First, tissue surface tension and contact angles are directly measured, and therefore

$\sigma' = \sigma$ (S3), $\theta' = \theta$ (S4), and $\theta_i' = \theta_i$ (S5).

For the free energy per cell at gap surfaces, β_f is replaced by $\beta_f' - W_g$. At the tissue surface, an elastic energy W_s is suggested by the surface of cell pairs being covered by a dense hyaluronan containing PCM and the pairs being dumbbell shaped as expected from a stiff surface (Parent et al. 2024), giving a free energy $\beta' - W_s$ instead of β. Thus,

$\beta' = \beta + W_s$ (S6), and $\beta_f' = \beta_f + W_g$ (S7).

Further, from a comparison of free energies at contacts $\beta_c = \beta_f - \Gamma/2$ and $\beta_c' = \beta_f' - W_c/2 - \Gamma'/2$, and from Eqn 4 and S7, that $\beta_f - B/2 = \beta_f + W_g - B/2 + W_g$ and thus B/2 is transformed as

$B'/2 = B/2 + 2W_g$ (S8).

Lastly, $\sigma_i' = (\beta_f' - W_g) - (\beta_f' - W_c/2 - \Gamma'/2)$ and with Eqn 4, $\sigma_i' = B'/2 - 2W_g$ and finally with Eqn S8, $\sigma_i' = B/2 + 2W_g - 2W_g$ and thus

$\sigma_i' = \sigma_i$ (S9).

With these transformations, respective differences and ratios of the respective free energies are (equations numbered as in main text)

$\sigma = (\beta' - \beta_f') + (B'/2 - (W_g+W_s))$ (5a), $\cos\theta = (\beta_f' - (B'/2 - W_g))/(\beta' - W_s)$ (5b),

$\sigma_i' = B'/2 - 2W_g$ (6a), $\cos\theta_i = (\beta_f' - (B'/2 - W_g))/(\beta_f' - W_g)$ (6b).

Finally,

$\alpha = 1 - \cos\theta_i$

$= 1 - [(\beta_f' - (B'/2 - W_g))/(\beta_f' - W_g)]$

$= [(\beta_f' - W_g) - (\beta_f' - (B'/2 - W_g))]/(\beta_f' - W_g)$

$= (B'/2 - 2W_g)/(\beta_f' - W_g)$

$= \sigma_i'/(\beta_f' - W_g)$,

confirming that α represents a dimensionless relative adhesiveness at gaps, as in the capillarity-based model. It takes on the same value since $\theta_i' = \theta_i$ and $\alpha = 1 - \cos\theta_i = 1 - \cos\theta_i'$.

References

Kabedev, A. and Lobaskin, V. (2018). Structure and elasticity of bush and brush-like models of the endothelial glycocalyx. *Sci. Rep.* **8**, 240.

Development • Supplementary information
